# Short read DNA fragment anchoring algorithm

**DOI:** 10.1186/1471-2105-10-S1-S17

**Published:** 2009-01-30

**Authors:** Wendi Wang, Peiheng Zhang, Xinchun Liu

**Affiliations:** 1 Institute of Computing Technology, Chinese Academy of Sciences, Beijing, 100190, PR China

## Abstract

**Background:**

The emerging next-generation sequencing method based on PCR technology boosts genome sequencing speed considerably, the expense is also get decreased. It has been utilized to address a broad range of bioinformatics problems. Limited by reliable output sequence length of next-generation sequencing technologies, we are confined to study gene fragments with 30~50 bps in general and it is relatively shorter than traditional gene fragment length. Anchoring gene fragments in long reference sequence is an essential and prerequisite step for further assembly and analysis works. Due to the sheer number of fragments produced by next-generation sequencing technologies and the huge size of reference sequences, anchoring would rapidly becoming a computational bottleneck.

**Results and discussion:**

We compared algorithm efficiency on BLAT, SOAP and EMBF. The efficiency is defined as the count of total output results divided by time consumed to retrieve them. The data show that our algorithm EMBF have 3~4 times efficiency advantage over SOAP, and at least 150 times over BLAT. Moreover, when the reference sequence size is increased, the efficiency of SOAP will get degraded as far as 30%, while EMBF have preferable increasing tendency.

**Conclusion:**

In conclusion, we deem that EMBF is more suitable for short fragment anchoring problem where result completeness and accuracy is predominant and the reference sequences are relatively large.

## Background

The emerging next-generation sequencing method based on PCR technology boosts genome sequencing speed considerably, the expense is also get decreased. It has been utilized to address a broad range of bioinformatics problems including: gene re-sequencing, polymorphism detection, small RNAs analysis, transcriptome profiling, chromatin remodelling, and etc. Limited by reliable output sequence length of next-generation sequencing technologies, we are confined to study gene fragments with 30~50 bps in general [1] and it is relatively shorter than traditional gene fragment length. For example: In [2], researchers used sequences in 2 K~100 Kbps range for gene alignment algorithm study. Genome query algorithm studied in [3], is based on 600 bps gene fragment in average. So we cannot use those older assembly or query algorithms on short-read sequences directly [4]. On the other hand, because of inefficiency, those existing algorithms cannot fully explore the high-throughput capability of next-generation sequencing devices. To illustrate the existing gap between raw data generating and processing speed, we take the throughput capability of Genome Analyzer System from Illumina for evaluation [1]. Meanwhile, we conservatively presume that the covering factor for re-sequencing process is 20. The net output sequence size would be 30 Gbps in single read mode (60 Gbps in paired read mode) for human gene. To evaluate the up-to-date processing speed, we use the 134 s/5 Mbps speed data from SOAP [5], also assume that this speed could be scaled linearly. By brief calculation, there will be at least 134*30 Gbps/5 Mbps = 228.7 CPU hours to match the raw data output capabilities!

Anchoring gene fragments in long reference sequences is an essential and prerequisite step for further assembly and analysis works. Due to the sheer number of fragments produced by next-generation sequencing technologies and the huge size of reference sequences, anchoring would rapidly becoming a computational bottleneck [6]. Also the accuracy and completeness of anchoring results would influence the quality of assembly result drastically. Basically, to solve the anchoring problem, we need to address three issues: (1) Error-tolerant strategies should be included. As a result, the candidate hit space will get amplified. Properly filtering out false-positive positions is the key to achieve high accuracy and speed; (2) For short-read sequences, new query paradigm should be devised to replace de facto "Seed-and-Extend" paradigm; (3) Deal with possible system degradation caused by huge data size or query count.

In this paper we divided sequence anchoring work into 2 phases: first an index structure based on frequency transformation was used to rule out most unqualified searching areas; in phase 2, an accurate matching process based on simplified Smith-Waterman algorithm[7] (SW for short) was used. The rest of the paper is organized as following: the remaining of this section will introduces some related works on gene sequence query algorithms. We introduce our algorithm in methods section, including how to identify differences between sequences and how to build index structure efficiently. We give experiment data to evaluate the performance of our algorithm in results section and followed by conclusions and future works as final section.

As gene sequences could be expressed as readable strings, lots of common string matching algorithms [8] could be used directly to solve the gene sequence query problem. In order to improve efficiency, sensitivity or accuracy of the baseline solution, quite a lot of research works have been done [9-12]. In general, we could categorize the sequence query problems into k-NN and range query [13]. If we care about highly identical results only, k-NN query would be helpful, where the query process could terminate after finding enough results of interests. In range query, the executing time and result accuracy could be fine-turned by initial parameters to suit for wide range of applications.

The various requirements from different bioinformatics applications result in performance and implementation divergence between different query algorithms. When data set and query count are relatively small, the traditional brute-force algorithms [7,14] could bring all needed data into memory, thus acceptable performance could be achieved without pre-processing work. However, the complexity of those algorithms will become intractable when problems size and query count get increased. By pre-processing the reference sequences and build fast searching index structure, we could avoid those unnecessary traverses of all data in each query request. To handle the index explosion [15-17], compression based indexing techniques are introduced in [18-20]. In [21], based on frequency and wavelet transformation, the researchers devised a multi-dimensional indexing method for fast sequence similarity search in DNA and protein database. On the other hand, when the query sequences remain unchanged, or we want to detect specific patterns in reference sequences, pre-processing the query sequence as well could be used to improve performance, such as HMM [22,23], FSM [17], suffix-tree [24] methods.

Best performance could be delivered by separating query work into two phases: approximate filtering and accurate matching. And it has been utilized by most query algorithms recently. The essential reason behind this method is that filtering work is relatively simpler than matching one, however with some degradation of result accuracy. Fast ruling out unqualified areas by filtering work, the workload passed to matching phase could be greatly reduced. Furthermore, we could transform the filtering work to frequency space problem, and make balance between efficiency and accuracy of different transforming mechanism. The matching phase could also be accelerated by converting it to sorting [18], best seed generating [25], covering and error rate model [19], approximate string matching [17], longest common substring [26] or other equivalent problems to solve.

On the other hand, usually researchers are concerning about the sensitivity and error rate of query results. We could evaluate the sensitivity as the completeness of result; it indicates that if all quantified positions could be found. And the error rate is antonym of accuracy; it could be expressed as if there have any false-positive positions in output results. These parameters would become fluctuated under different query workloads. By introducing scoring matrix to measure difference between bio-sequences, like BLOSUM [27], PAM [28], suitable matrix could be used for specific applications in order to get high sensitivity and low error rate; some algorithms, as SSAHA [3], MRS [13], Pattern Hunter [25], resort to find a biological independent and generalized algorithm. The sensitivity and error rate are differed from one and another; for the other algorithms, as BLAT [18], IDC based method [19], the output result is deeply influenced by the similarity between input sequences. To get expected sensitivity and error rate, these methods require input sequences comply with certain restrictions.

The research area for short-read sequencing technology is relatively new, however there already have some basic achievements. In [4,29], short read sequence alignment algorithms are devised. Also, there exists some solutions to solve short read sequence anchoring problem, as Maq [30] could handle 2~3 miss match error; SOAP could handle either 1~3 continuous gap error or 1~2 miss match errors in querying and aligning problems.

## Methods

The algorithms studied in this paper could be expressed as range query with error tolerance of 2 miss match or 1 gap, and is dedicated to Illumina-Solexa sequencing technology. The sequence errors are largely incurred by equipment and experiment process fault, as the high per base read accuracy (> 98.5%) given in [1], considering arbitrary errors would be unnecessary. Because of those included errors, during comparing process, if the two sequences under test cannot be identified as equal, measuring metrics should be established to capture their difference. Instead of doing the time consuming char-by-char comparison work directly, we could transform given string into multi-radix frequency vector, and using various vector approximation or compression methods to simplify comparing cost [15]. In following section, we will use 8 bps fixed window length to sample reference sequences and then transform those extracted substrings to correlated frequency vectors over a 4-dimentional frequency space. Also an Euler distance is introduced to capture vector variation in this space.

### Frequency transforming

As there would require at least 4^k ^operations to calculate the distance between two k-gram frequency vectors. The frequency transforming used in this paper is confined to 1-gram semantics [13]. Because only in this way could we get the expected simplification for comparing work. For a given sequence S = s_1_s_2 _... s_n_, let |S| = n donate the sequence length, and express the alphabet of the sequence as ∑ = {a_1_, a_2_,..., a_m_}. We define a frequency vector F = {f_1_, f_2_,......, f_m_} for each sequence. The elements of F satisfy relationship expressed in equation (1):

(1) $$\begin{array}{cccc} i) & 0\leq f_{i}\leq n & \text{ii)} & \sum_{1\leq \text{i}\leq \text{m}}f_{i}=n \end{array}$$

The definition of Euler distance is relatively simple as following.

**Definition 1 **The Euler operation on a m-radix vector V = {v_1_, v_2_,..., v_m_} is to add another equal dimensional vector C = {c_1_, c_2_,..., c_m_} on it.

**Definition 2 **If a frequency vector V = {v_1_, v_2_,..., v_m_} complied with equation (1) we say it is valid; If a m-radix transforming vector C = {c_1_, c_2_,... c_m_} complied with equation (2) we say it is valid.

(2) $$\begin{array}{ccc} \sum_{1\leq i\leq m}c_{i}=0; & \sum_{1\leq i\leq m}|c_{i}|=2; & c_{i}\in \{-1,0,1\}; \end{array}$$

**Definition 3 **An Euler operation on a valid frequency vector F = {f_1_, f_2 _..., f_m_} is valid, if the result vector F' = {f'_1_, f'_2_,..., f'_m_} is still valid.

In order to maintain the validity of Euler operation, we need to restrict the content of transforming vector C in theorem 1.

**Theorem 1 **For valid transforming vector C and valid frequency vector V, if c_i _= -1 then v_i_! = 0 holds for each element in C and V, then after apply vector C on V, the result vector is valid.

**Proof: **C is valid, so there have only 2 non-zero elements in C, note as c_i _= 1, and c_j _= -1. After Euler operation we get result vector V', where only two elements differ from V as: v'_i _= v_i_+c_i _= v_i_+1, v'_j _= v_j_+c_j _= v_j_-1. Because v_j_! = 0, v_i _< n, we get 0 ≤ v'_i_, v'_j _≤ n and ∑v'_i _= ∑v_i_+1-1 = ∑v_i_. As V is valid so ∑v_i _= n holds, we get ∑v'_i _= n. According to definition 2, V' is valid.

**Definition 4 **We call two valid frequency vector V_1 _and V_2 _are similar, if |V_1_| = |V_2_| and ∑V_1 _= ∑V_2 _holds.

**Definition 5 **The Euler distance between two similar frequency vectors is defined as minimal Euler operation required to transform one frequency vector into the other.

We could use equation (3) to calculate Euler distance between two similar vector U and V.

(3) $$ECD(U,V)=\sum_{1\leq i\leq m}\frac{\left|u_{i}-v_{i}\right|}{2}$$

Until now, we haven't considered gap errors when building transforming vectors yet. The gap errors would incur the offset of consecutive sequence, so it contradicts with the method introduced in this section where accurate positional information is used. However, in following section, this problem could be solved properly by a block-reading technique with initial offset.

### Blocked frequency transforming

Although by calculating Euler distance between two frequency vectors, the time-consuming char-by-char comparing work could be avoided. However, after the converting work, certain positional information will get lost. Moreover the sampling window length is restricted by the total sequences length we studied in this paper, so we devised a novel way to pre-processing reference sequences: firstly, the original sequences were divided into blocks, and then frequency transforming was taken on each individual block, finally we using 4 consecutive blocks to build a 4-dimensional bounding space similar as the 2-dimensional MBR given in [13]. It's clear that the positional information between blocks is maintained, while with some information loss within each block. The Euler distance between query and reference sequences is calculated by sum the 4 block's ECD value respectively. Next, we introduce valid partition concept for dividing gene sequence into blocks.

**Definition 6 **The partition result of a given sequence S = s_1_s_2 _... s_n _is a set of blocks B = {b_1_, b_2_,..., b_k_}. If B satisfies following conditions: (i) For any element s_i _∈ S, there have and only have one block say b_j _in B, so that s_i _∈ b_j_. (ii) All elements in B are nonempty. We say this partition B is valid.

**Definition 7 **For a valid partition B, if the covering rate keeps above p with any drop of ε blocks, we say B is a ε-p partition.

For example if we want to build an index structure with 16 bps entry on 32 bps input sequences, and want to tolerate 2 arbitrary errors. It's needed to give a 2-0.5 partition, so that when there have 2 arbitrary errors, we still have 32*0.5 = 16 bps accurate characters to use as accurate sequence to retrieve index structure. When using fixed-length and non-overlapping sample window, table 1 gives the comparison of coding length and compression rate between binary coding and vectorized coding styles. It's clear the when increasing sample window length, compression result will get improved, but with more data loss. To evaluate general filtering effect, however, quite a lot of factors should be considered as: similarity between sequences, frequent transforming strategies, the length of sample window, and etc [21]. In the remaining part we will set the sample window and blocking length to 8 bps for simplicity.

**Table 1:** Coding results for variable sampling window length.

**Sample window length**	**1**	**2**	**3**	**4**	**5**	**6**	**7**	**8**	**9**
Vector count	4	10	20	35	56	84	120	165	220

Binary coding length	2	4	6	8	10	12	14	16	18

Vector coding length	2	4	5	6	6	7	7	8	8

Compression rate	0%	0%	16.7%	25%	40%	41.7%	50%	50%	55.6%

The compression rate is calculated as the difference between binary coding length and vector coding length divided by binary coding length. The vector count is calculated as C(w+m-1, w) where w is the sampling window length, m is the size of alphabet used to form the sequences. The vector coding length is the minimum value n which let 2^n ^> vector count holds.

### Filtering and matching algorithm

Before give out our algorithm, we make formal definition of filtering and matching problem first.

**Definition 8 **For restriction p ≥ 0, assume that S could be divided equally into n blocks with equal length. If there have at least n-p blocks which have one-by-one mapping relationship with n-p blocks within the other sequence T, then we say S, T has hit relation under restriction p.

**Definition 9 **For restriction G ≥ 0, M ≥ 0, if sequence S and T satisfy either of two following conditions: (1) If there have G_1 _gaps in S, G_2 _gaps in T, and G_1_+G_2 _≤ G. The remaining min{|S|-G_1_,|T|-G_2_} positions in S and T are identical. (2) If there have M miss matches in S and T, the remaining min{|S|-M,|T|-M} positions in S and T are identical. We say that S, T has match relation under restriction G and M.

**Definition 10 **Give sequence S and T, and assume that |S| > |T|, set maximum tolerated miss match errors to M, and maximum tolerated gap errors to G. The filtering problem is to find any offset i in S, so that S [i, i+|T|-1] and T have hit relation under restriction max(M, G).

Similarly, we could define the matching problem as following, and theorem 2 explains the correlation between filtering and matching relationship.

**Definition 11 **Give same conditions as in definition 10. The matching problem is to find any offset i in S, so that S [i, i+|T|-1] and T have match relation under restriction G and M.

**Theorem 2 **For sequence S and T, hit relation is a necessary condition for their matching relation.

Proof: Assume that S and T have matching relation, however don't have hit relation. According to definition 8, for restriction p = MAX{G, M}, the number of blocks in S and T which have one-to-one correspondence will less than n-p, namely the miss match block number q will large than p. When those unmatched blocks was caused by gap errors, as G_1_+G_2 _= q > p = MAX{G, M} ≥ G, we get G_1_+G_2 _> G. Similarly, when those unmatched blocks was caused by miss errors only, we will get q > M. It contradicts with definition 9, so the assumption is incorrect, and the theorem holds.

**Figure 1:**
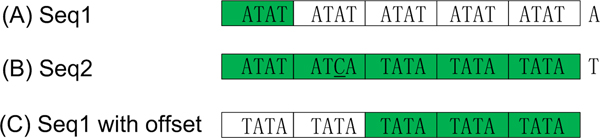
**Blocking strategy with initial offset**. As shown in part A and B, seq1 and seq2 are divided into 5 blocks containing 4 bps each. The gap error caused by missing of character C at 7^th ^position in seq1 made it fail to match with seq2. However, as show in part C with additional reading frame for seq1 with 1 bp shift left. We could collect enough matching blocks (highlighted with dark background) to deduce the hit relationship.

Now we consider how we could solve arbitrary gap errors. For N-bps sequence, when partition it equally into m bps blocks, we get N/m = n blocks. One arbitrary miss error would contaminate 1 block at most, so for p miss matches; there still have n-p accurate blocks to deduce hit relationship in definition 8. However, one gap error would contaminate all its consecutive neighbours. Figure 1 illustrated a sequence reading method with initial offset which could be used to solve gap error. Generally, to tolerate G arbitrary gap errors, we need to consider G+1 reading frames; however when using blocking method, only L-1 arbitrary gap errors could be tolerated, where L is the length of sampling window.

The EMBF (Euler-distance Mapping based on Block Filtering) algorithm is given in table 2. The kernel of the EMBF algorithm is a two-level index structure. Different combination of blocks is used as address to access a map-liked index structure. The output (usually a pointer or block set number) is used to retrieve continuous blocks in second level index. Then we calculate Euler distance on different blocks, Euler distance of a sequence is represented as the summation of Euler distances of its sub-blocks. Notice that, we could terminate the distance calculation; if the summation up to one block is already exceeds the predefined threshold.

**Table 2:** Procedure of EMBF algorithm.

**Input: Block length L, n bps reference sequence S, m bps query sequence T, miss match error threshold M, gap error threshold G.**
Let B1 = ⌊*n*/*L*⌋, B2 = ⌊*m*/*L*⌋, E = MAX(M, G);
**1. For offset = 1 to L do**
1.1 Divide S [offset, n] into L bps blocks, as S_offset _= {s_offset,1_,..., s_offset, B1_};
1.2 Convert S_offset_to frequency vectors, as ES_offset _= {es_offset,1_,..., es_offset, B1_};
**2. For offset = 1 to L do**
2.1 Sequentially choose B2 blocks from ES_offset_, and set the start position as p; Using all possible combinations to get B2-E blocks. And combine them as ADDR variable. Set the remaining E blocks as r;
2.2 Mapping pair (ADDR,(r, p)) into a hash map M, and chaining possible conflicts;
2.3 Iteratively scan ES_offset _for next B2 blocks in ES_offset_;
**3 First level filtering process**
3.1 Divide T into L bps blocks, as T = {t_1_, t_2_,..., t_B2_} and convert them to frequency vector as ET = {et_1_, et_2_,..., et_B2_};
3.2 Choose B2-E blocks from ET and combining them as ADDR variable, set the remaining E blocks as t;
3.3 Query ADDR in M and pass all returned results as R = {(r_1_, p_1_), (r_2_, p_2_),...... } to step 4;
**4 For i = 1 to | R| do**
if ECD(t, r_i_) < E then record p_i_;
**Output: All recorded p_i _from step 4.**

The step 1~2 in EMBF are pre-processing steps where a two-level index structure was constructed. Index entry addresses are generated according to different combination of blocks, and require L*n/L*C(m/L,2) = nm^2^/L^2 ^operations in total. The computing overheads to generate ADDR could be set as constant c, so the total pre-processing costs to build the index is cnm^2^/L^2 ^≈ O(n). Step 3 is the first level filtering phase with constant computing cost. The output result count is related to the length of index seed and the size of reference sequence. The second level filtering work is processed in step 4 with time complexity of O(rm/L), where r is the average output count of step 3, see results section for accurate evaluation of the value r. So by excluding the pre-processing steps, the timing complexity of EMBF is O(rm/L) << O(n). The space complexity could be interpreted as memory space used to implement the two-level index structure (see results section for detailed analysis). In order to fit first index into fast storage device to achieve best performance, we could adjust the size of reference sequence and the length of index seed to fine tune the index size and access overheads.

**Figure 2:**
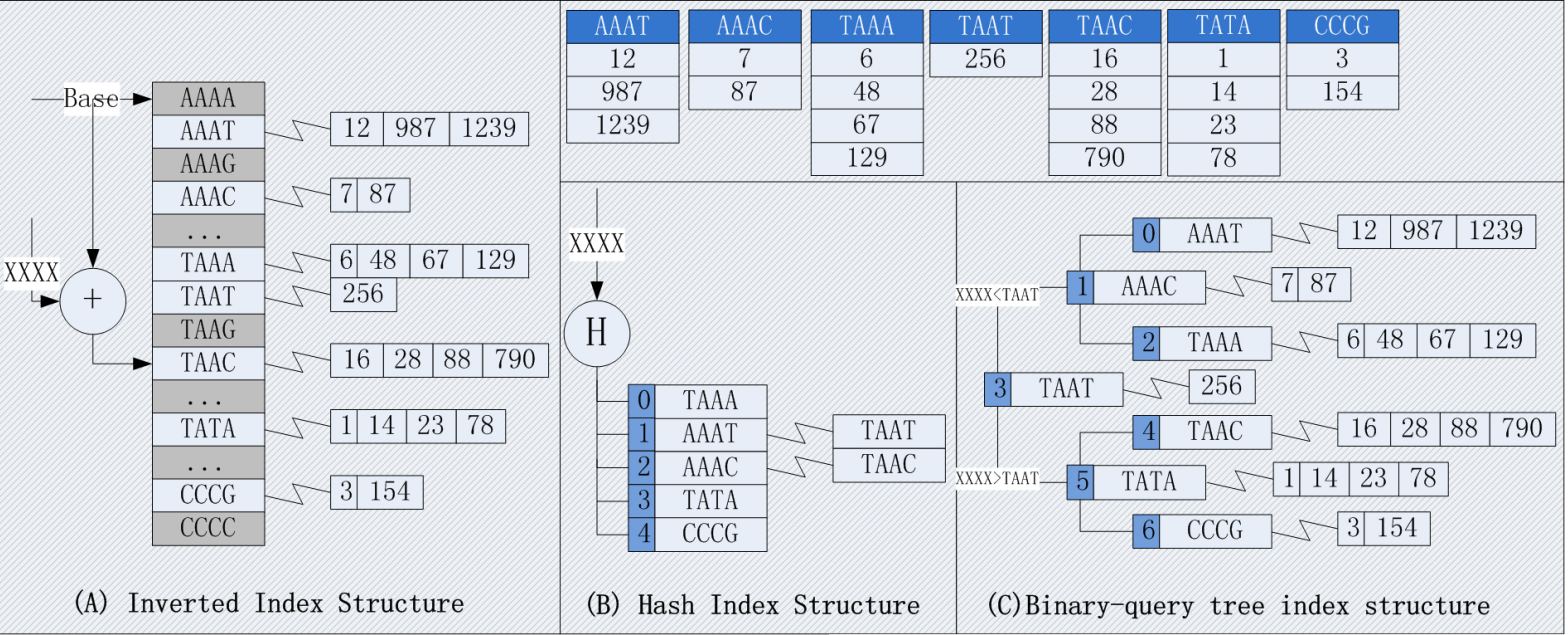
**Three difference index structures**. The numbers in right-up part of the figure gives the offset in reference sequences where the given sequence fragment have identical occurrence. In part A, blocks with dark background indicates placeholder where no actual data exists. In part B, a hash function H is performed to hash input sequences into buckets labelled with 0~4, possible conflicted sequences are chained together. Part C illustrates a binary search tree, and the number at the beginning of each block is used as the search key.

We studied 3 different index structures given in figure 2. Taking the sample blocks in up-right corner for example, we could organize them into an inverted-index structure as show in figure 2(a). The content of a block is used as an offset to shift a base address to access a continuous array. If query block have occurrence in the reference sequence, its first position will be stored in the array element with the other positions followed. The query process is considerable simple and efficient for inverted-index, however the wasted storage is also considerable, as shown in figure 2(a) the utilization rate of this example is only 7/256. Meanwhile, it's not suitable to build index structure for long sequences. In figure 2(b), hash method was used to distribute blocks into different storage locations. Besides providing efficient hash algorithm; we should solve the increased overheads caused by long conflict chaining. The n-radix query tree could also be used to organize index structure as illustrated in figure 2(c), the challenges lies in how to overcome building overheads and explore efficient parallel query algorithm. We have chosen the hash strategy for implementation considering their efficiency and simplicity. The other two structures will be studied further in future work.

The filtering output results will have some false-positive errors, so detailed matching phase is needed to refine those raw results. The difference on total length between query and reference sequence is oblivious, also the expected arbitrary errors in each reading frames are also limited. A simplified version of SW algorithm which only consider those leading diagonal and some sub-diagonals are already efficient enough. For example, to tolerate G gap errors, by transposing the scoring matrix, we could confine our query space to G+1 diagonal in upper/lower triangular score matrix. Compared with systolic array, the computational complexity is optimized from O(n^2^) to O(Gn+n), the space complexity is improved from O(n) to O(G+1).

### Fine-grained parallelization

The executing cost of different part of EMBF under various working set is listed in table 3. When the working set gets increased, the overhead introduced by filtering phase will become the dominant one; the increment of time cost in percentage from 38.93% to 63.78% properly justified this phenomenon. At the same time we cannot ignore the overheads caused by matching phase, so it's needed to accelerate those two parts simultaneously. We could simply add parallel matching units to solve the contentions caused by sequential matching. Moreover, as there do not have data sharing relationship between different parts of reference sequences, it's expected to get linear scalability. For the filtering phase, in order to increase data locality, we divided large reference sequences into smaller chunks, and built structured index for each chunk individually. Thus the unnecessary data sharing overheads caused by big centralized index structure is eliminated. Those pre-calculated small index structures could be stored in an index pool. Those index structures are downloaded to different parallel processing units at runtime. After the calculation, a result collecting unit will gather output results and upload it to higher level of system. In filtering unit, further fine-grained parallelism could be explored, as we could divide the index structure by different block combinations as explained in EMBF algorithm, and do filtering work concurrently on different block combination. By eliminating those unnecessary data sharing, embarrassingly parallel possibility would be expected.

**Table 3:** Executing time analysis of EMBF

**Data Set**	**Filtering**	**Matching**	**Addressing**	**Others**
**33.7 Mbps**	38.93%	42.13%	3.57%	15.37%

**69.3 Mbps**	41.00%	38.71%	3.06%	17.23%

**134 Mbps**	63.78%	22.41%	1.6%	12.12%

The filtering and matching column corresponds to time consumed in percentage for step 3 and step 4 in EMBF algorithm repetitively. We also separated the overhead to generate the index access address, and listed it in addressing column. The others column include sequence reading, results writing and some log utility overheads. The value in this table is the mean value of 10 K anchor executing results.

## Results and discussion

We used 4, 7, 11 and X human genome contig sequences from NCBI [31] to synthesize the reference data sets with total size of 33.7 Mbps, 69.3 Mbps, 134 Mbps and 359 Mbps each. The short read sequences were synthesized by randomly extract 32 bps fragments from each data set and insert arbitrary miss match or gap error into them. To rewrite synthesized sequence we introduce 1 miss match with possibility of 8%, 1% for 2 miss matches and 1% for 1 gap. The remaining 90% are left untouched. The SW algorithm, BLAT and SOAP algorithms are tested against EMBF to compare their performance. In order to eliminate possible infection caused by pre-processing and warm-up step, only the computing kernels are profiled blow.

The BLAT and SOAP algorithms have a broader error tolerant capacity than EMBF does, so we carefully adjusted the input parameters for BLAT and SOAP in order to minimize this influence. For example we set the tile size in BLAT to 10 bps, and using the ooc tag to enable the masking strategy for overused tiles introduced in BLAT, also the maximum gap between tile was set to 1; for SOAP 12 bps seed was used, it is set to scan both chain and output all hit results, also the allowed miss match and gap errors were set to 2 and 1 respectively. The memory utilization was largely due to the space cost to implement different index structures, which will be analyzed in following section.

### Index structure overheads

**Figure 3:**
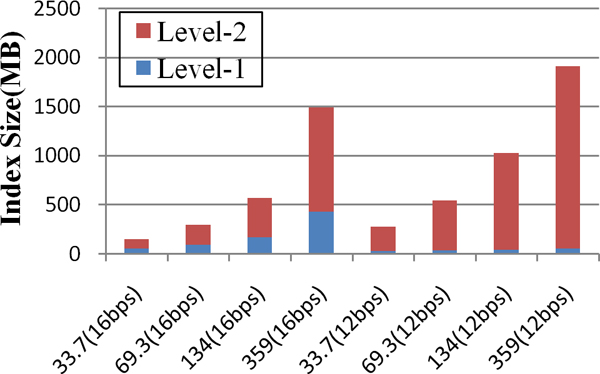
**Memory cost of EMBF**. The data was collected from 33.7, 69.3, 134 and 359 Mbps data set respectively. To evaluate the influence of different seed length 12 bps and 16 bps seed was tested.

The memory consumption of different index structure under 33.7 Mbps dataset is listed in table 4. EMBF uses the hash index as shown in figure 2(b), while SOAP uses inverted-index structure as shown in figure 2(a). Although only 12 bps index seed length is used in SOAP, the memory consumption is already 2 times when compared with EMBF-12 bps. The concepts of BTree index is similar with n-radix query tree as shown in figure 2(c), it's clear that there do not have memory consumption advantages for BTree when compared with SOAP and EMBF. In figure 3 we compared memory consumption of EMBF when the dataset size is varied. Because of the inherent clustering property of gene sequences, although the first level index could be compressed when decrease index seed length. However, the second level index will considerable increased as more and more positional information need to be stored. We set seed length to 16bps for EMBF, in order to balance the size of the two-level index.

**Table 4:** Memory consumption to implement index structure (MB).

**Index name**	**First-level**	**Second-level**	**Total**
**EMBF-12 bps**	28.24	247	275.24

**EMBF-16 bps**	49	99	148

**BTree-11 bps**	176	397	573

**BTree-16 bps**	342	397	739

**BLAT**	-	-	60

**SOAP**	-	-	562

We divide the memory consumption for EMBF and BTree to two separate parts, the first part is used to build a hash map for EMBF and a traversal query tree for BTree; the second part is used to store positional information for EMBF and remaining sequences for BTree. The -xbps suffix in index name column indicates that the algorithm using seed with length of x bps.

### Filtering result analysis

**Figure 4:**
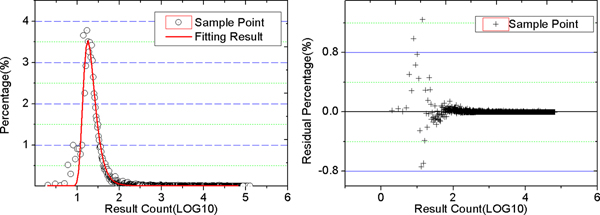
**Filtering results of 10 K query on 359 Mbps dataset**. We collected filtering results by anchoring 10 K synthesized sequences on 359 Mbps dataset. The maximum of percentage (3.528%) occurs when x = 1.251, the correspondent filtering result count is 17.834. The residual percentage is well below ± 0.8%, which indicates that the output result count in step 3 of EMBF comply with Gumbel extreme distribution.

To evaluate the quality of filtering result, we fit the discrete output result count with Gumbel extreme distribution [32]. Figure 4 gives the fitting curve and residue analysis, and result could be expressed as equation (4). By integrating this equation, we calculated the ceiling probability for different output count value. For example: the probability that the output count is less than 7205.8 is 99%, less than 63.9 is 95%, less than 42.01 is 90%.

(4) $$\begin{array}{l} y=y0+A*\exp(-z-e^{-z}+1)\\ z=(x-xc)/w\\ y0=0.014;xc=1.251;w=0.138;A=3.528 \end{array}$$

### Performance analysis

In table 5 we listed the relative speedup. The results are collected by using SW, EMBF, SOAP and BLAT separately to execute the same 10 K query on 35.7 Mbps dataset. To explain the speed advantage of SOAP, we need to notice that only 3 of the 6 possible block combinations are used to build index structure in SOAP, thus the total workload did in SOAP is actual 1/4 of what EMBF did. The consequence is that lots of match positions will get lost in SOAP; similar problem also exists for BLAT, especially when enabling the over-occurrence tile filtering property. It is assumed that the output of SW is accurate and complete, so could be used as reference to quantify other algorithms. As shown in table 6, the output result of EMBF is identical with SW, however, the output result of SOAP and BLAT is far from satisfaction. We also implemented a simplified version of EMBF, the EMBF-3#, where only 3 of the 6 possible block combinations are used as SOAP did. So we say EMBF have advantages on results accuracy and completeness over the others.

**Table 5:** Relative speedup comparison.

**Speedup**	**EMBF**	**EMBF-3#**	**SW**	**BLAT**	**SOAP**
**EMBF**	1	1/1.57	48838	42.66	1/3.1

**EMBF-3#**		1	76734	67.02	1/1.97

**SW**			1	1/1145	1/151385

**BLAT**				1	1/132.3

**SOAP**					1

The value indicates the speedup when comparing row algorithm with column algorithm. A value n > 1 means that the row algorithm performs n times fast than column algorithm. All data were collected from average performance of 10 K anchor requests.

**Table 6:** Result accuracy comparison.

**Algorithm**	**33.7 Mbps**	**69.3 Mbps**	**134 Mbps**
**SW**	202676	300375	NO DATA

**EMBF**	202676	300375	1433261

**EMBF-3#**	129930	198788	900084

**SOAP**	24544	47202	107297

**BLAT**	44298	77891	217973

**BLAT-OOC10**	42907	76840	213766

The NO DATA indicates that the executing time to get final result was so long, which will be ignored in this paper.

### Efficiency and scalability analysis

**Figure 5:**
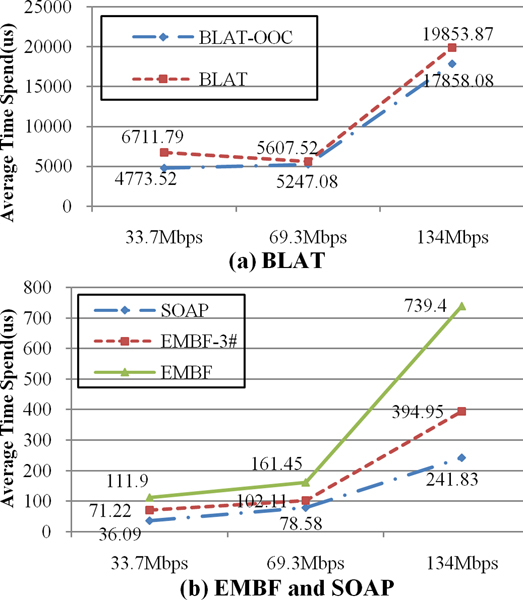
**Scalability analysis**. BLAT with ooc tag enabled will have a better performance, but the completeness of output result will get degraded. The average value of 10 K anchor request was used to smooth out jitter and vibration of individual query request.

The share-nothing relationship between different parts of reference sequences made the scalability analysis of EMBF algorithm simplified. By applying the divide-and-conquer methods, only single node scalability needs to be tested. In figure 5, average querying time consumed by BLAT, EMBF and SOAP on different data set is given. When reference sequences get increased, they both suffer from performance degradation. This phenomenon also justified the conclusion given in previous section that we need to separate large centralized index into smaller distributed ones, in order to overcome possible high access and sharing overheads. Also the SOAP algorithm have some speed advantages over EMBF, however it's based on great accuracy loss as illustrated in table 6. They both outperformed BLAT for 25~200 times.

**Figure 6:**
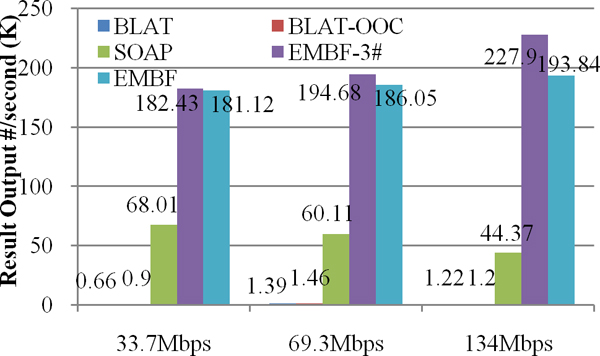
**Efficiency comparison**. Efficiency is defined as total output result count divided by total time consumed. The data show that EMBF have 3~4 times efficiency advantage over SOAP, and at least 150 times over BLAT.

In figure 6, we compared algorithm efficiency for BLAT, SOAP and EMBF. The efficiency is defined as output result count divided by total time consumed. The data in figure 6 show that EMBF have 3~4 times efficiency advantage over SOAP, and at least 150 times over BLAT. Moreover, when the reference sequence size is increased, the efficiency of SOAP will get degraded as far as 30%, while EMBF have preferable increasing tendency.

## Conclusion

By defining a gapless Euler distance and a sequence reading technique with initial offset, we introduce a frequency transforming method based on fix-length blocking mechanism. In our approach, the filtering phase could considerably alleviate the workload passed to the time-consuming matching phase, and in turn those false-positive results caused by inaccuracy of filtering process could be further refined. In order to accelerate filtering speed, a two-level index structure based on hash method is developed. By adjusting input parameters, as index seed length and the size of reference sequences, we could trade off between implementation and query overheads to get optimized performance. We also show that to avoid the unnecessary data sharing, a large centralized index structure could be divided to smaller distributed ones, which is much more suitable for massive parallelization. Efficiency of EMBF algorithm is 3~4 times better than up-to-date fastest one, while with comparable executing overheads. Moreover when problems size gets increased, the efficiency of EMBF have preferable increasing tendency. Also EMBF was devised for short sequences, where the length is usually around than 30~50 bps, when the length of query sequence get increased we could use enlarged sampling window length to make it more adaptive, however their need further experiments to evaluate efficiency of EMBF under different input sequence length, which will be list as future work.

In conclusion, we deem that EMBF is more suitable for short sequence anchoring problem where result completeness and accuracy is predominant and the reference sequences are relatively large. The future work includes: developing of specialized hardware devices to accelerate the index access, exploration and implementation of fine-grained parallelism, index compression, revise the algorithm to consider arbitrary errors and input length.

## Competing interests

The authors declare that they have no competing interests.

## Authors' contributions

WDW carried out algorithms design, and drafted and revised the manuscript; he also gives the design of the experiment and performed the result analysis. PHZ participated in the alignment algorithms design and evaluation, he also helped to revise the manuscript. XCL participated in the alignment algorithms design and evaluation. All authors read and approved the final manuscript.
